# A randomised feasibility trial comparing group and individual format GROUPS FOR HEALTH interventions for loneliness in people who experience psychosis

**DOI:** 10.1111/papt.12574

**Published:** 2025-01-29

**Authors:** Lorna I. Hogg, Laura G. E. Smith, Catherine Haslam, Lyndsay Coxhill, Tim Kurz, Georgina Hobden, Anthony P. Morrison

**Affiliations:** ^1^ Department of Psychology University of Bath Bath UK; ^2^ Oxford Health NHS Foundation Trust Oxford UK; ^3^ Harris Manchester College University of Oxford Oxford UK; ^4^ School of Psychology University of Queensland Saint Lucia Queensland Australia; ^5^ School of Psychological Science University of Western Australia Perth Western Australia Australia; ^6^ Department of Experimental Psychology University of Oxford Oxford UK; ^7^ Division of Psychology and Mental Health University of Manchester Manchester UK; ^8^ Greater Manchester Mental Health NHS Trust Manchester UK

**Keywords:** empathy, G4H, identity integration, internalised stigma, loneliness, psychosis, social identity, wellbeing

## Abstract

**Objectives:**

Loneliness in people who experience psychosis is common and associated with poor mental health. In this randomised trial, we tested the feasibility and acceptability of an adapted groups for health (G4H) intervention for loneliness, delivered in group or individual format.

**Design:**

Mixed methods, two‐arm feasibility randomised controlled trial.

**Methods:**

Forty individuals who self‐identified as having psychosis were recruited from UK mental health care services, recovery colleges and charities. G4H was modified for people with psychosis, with participants randomised to receive the intervention delivered via group (*N* = 20) or individual (*N* = 20) format. The primary outcomes related to trial acceptability and feasibility. Exploratory repeated measures ANOVAs and t‐tests evaluated differences between formats over time in loneliness, wellbeing and possible mechanisms of change including social identification, identity integration and perceived in‐group and out‐group empathy. Measures were completed at baseline, end of treatment and 1‐ and 6‐month follow‐up.

**Results:**

Recruitment, retention and trial acceptability ratings for both group and individual formats of G4H were acceptable to good. No participants reported experiencing a serious adverse event. Exploratory ANOVAs indicated no differences related to format but positive change in key variables of loneliness, wellbeing, social identification and identity integration over time. *T*‐tests for loneliness indicated that this change was step‐wise from baseline, through end of treatment to 1‐month follow‐up.

**Conclusions:**

G4H is a feasible intervention for people with psychosis who identify as lonely and it can be delivered in either group or individual formats. This feasibility trial provides support for a future full randomised controlled trial.

## INTRODUCTION

Psychosis is common, with a median lifetime prevalence of 7.49 per 1000 (Moreno‐Küstner et al., 2018). Psychotic experiences vary between individuals and can include hallucinations, delusions and disrupted thinking, speech and behaviour American Psychiatric Association (APA, 2022) all of which can affect social functioning. Rates of sustained social and clinical recovery for psychosis are poor (Jääskeläinen et al., 2013) and levels of social disability persist even with early clinical remission (Wiersma et al., 2000). Underlying attachment issues (Berry et al., 2019; Gumley et al., 2014) and social‐cognitive processes such as theory of mind challenges (Bora & Pantelis, 2013; Brüne, 2005) can undermine capacity to engage socially and increase risk of loneliness. Associated mental health problems such as social anxiety (Achim et al., 2013; Aunjitsakul et al., 2021; Cosoff & Hafner, 1998; Gumley et al., 2004; Pallanti et al., 2004), poor self‐concept (Brohan et al., 2010; O'Connor et al., 2018; Williams, 2008) and self‐stigma and low self‐esteem (Vass, 2016; Vass et al., 2015; Vass et al., 2017) can further compound these social impacts. Impaired social interactions, therefore, present one of the greatest challenges for people experiencing psychosis (Couture et al., 2006).

### Loneliness and psychosis

Difficulties with developing and sustaining relationships mean that loneliness in psychosis is common and an important target for intervention. Badcock et al. (2015) report 79.9% prevalence of loneliness in people with psychosis compared to 35% in the general population. Giacco et al. (2016) found 80% of people experiencing psychosis reported feeling lonely and only 30% had more than one social contact in the previous week. Such isolation is not only associated with lack of social support and poorer quality of life (Norman et al., 2005; Wang et al., 2018), but also poorer health outcomes, including depression and anxiety (Lim et al., 2018). The UK National Institute for Health and Care Excellence guideline for Psychosis and Schizophrenia recommends that clinical work with people experiencing psychosis include assessment of the domains of ‘psychological and psychosocial, including social networks’ and that care across all phases of recovery should include information and advice about ‘building a social network’ (NICE, 2014).

### Interventions for loneliness

Previous research interrogating loneliness interventions suggests that social network size can be increased (Anderson et al., 2015) and that psychological interventions are effective at reducing loneliness (Hickin et al., 2021; Mann et al., 2017; Masi et al., 2011). Interventions targeting social recovery for people with psychosis to date have largely employed an individual format and focus on increasing social activities as the mechanism of change; examples include SUPEREDEN (Fowler et al., 2018) and the social coaching programme, SCENE^1^ (Giacco et al., 2021). Interventions for loneliness with the strongest evidence are CBT‐informed (Hickin et al., 2021) or delivered in a group format (Cattan et al., 2005). G4H is one such group approach and aims to build a sense of belonging in the therapy group as a way of helping people develop confidence to optimise existing group ties and to extend and sustain group ties in ways that support ongoing health and wellbeing. It is supported by evidence from several randomised controlled trials (Cruwys et al., 2021; Cruwys, Haslam, Rathbone, et al., 2022; Haslam et al., 2019; Haslam, Cruwys, Haslam, Dingle, & Chang, 2016). In a randomised controlled trial of 120 adults presenting with social isolation and depression, G4H produced a greater reduction in loneliness than TAU (*d* = −1.04 vs *d* = −0.33) (Haslam et al., 2019). Similarly, the pre‐post effect size for loneliness in a phase 3 randomised non‐inferiority trial comparing G4H with CBT in a sample of 174 adults experiencing loneliness and clinically significant depression was *d* = −1.07 compared with CBT *d* = −.89 (Cruwys et al., 2021). Cruwys, Haslam, Haslam, et al. (2022) draw on the data from three such trials to consider feasibility and acceptability. They conclude that G4H has good retention (>80%), good compliance with homework (<10% reporting non‐compliance) and both therapists and clients stressed that the group context was important to derive benefit. Further, Cruwys, Haslam, et al. (2023) demonstrated from analyses of data across two trials that those with diagnosed mental illness or who have more severe baseline depression tended to have better treatment outcomes. Better attendance at sessions also predicted better outcomes. However, these results are in people with depression and social anxiety, G4H has yet to be evaluated with people who experience psychosis. This is important given people with psychosis are a unique population with challenges around social relationships and stigma that might compromise their ability to engage in a group‐based intervention.

### Underlying psychological mechanisms

Understanding the psychological mechanisms that underpin both loneliness and psychosis may facilitate development and implementation of targeted therapeutic initiatives. G4H is informed by social identity theorising and its application to health (SIAH; Jetten et al., 2009) and therefore might be a particularly helpful approach for people who experience psychosis.

Fundamental to the social identity theorising underpinning G4H is the idea that group memberships are an important part of one's identity, that is, the sense of ‘us’ and ‘we’ that comes from belonging to family, friendship, work, community and interest groups, for example. Group memberships such as friendship groups support health and wellbeing through providing both social resources and a feeling of positive distinctiveness. When a positive source of influence, these group memberships can build self‐esteem, provide a basis for support and help people to feel more in control of their lives. These are particularly important in periods of challenge, adversity and life change such as when developing a new health condition such as psychosis. The G4H program helps people to realise the value of social networks and resources and to manage these more effectively so they can be accessed as needed when facing challenges that can undermine health. Thus, G4H can be viewed as a social identity management program.

Strategies that allow people to expand their number of social identities, and develop and integrate a positive social identity as someone who has psychotic experiences with other important social identities should improve social connectedness and thereby reduce loneliness and improve wellbeing. Consistent with this, frequency of social interactions with friends is associated with clinical recovery in psychosis (Bjornestad et al., 2017). Further, the potential mechanisms of in‐group identification, that is identification with others who also experience psychosis, and the integration of this identity with other important social identities into a coherent self‐concept, are associated with wellbeing in voice hearers (Hogg et al., 2022; Sheaves et al., 2021). Additionally, Hogg et al. (2023) found that perceiving other voice hearers to be empathic was associated with identification as a voice hearer, and perceiving those who do not themselves hear voices to be empathic was associated with the integration of a voice hearer identity with other important social identities. By implication, this suggests that perception of empathy in similar others is likely to facilitate the social identification process and this might be expected to be facilitated in the group format of the G4H intervention. Previous research found G4H identification and multiple groups belonging to be mechanisms of change (Haslam, Cruwys, Haslam, Dingle, & Chang, 2016). Perception of empathy in those who do not identify as having psychosis should facilitate the identity integration process. In this way, selectively joining social groups comprising individuals who are empathic towards people who experience psychosis may be important for developing a coherent and positive sense of self and improved wellbeing. Key questions then become how best to intervene to achieve these outcomes and whether a group format, as opposed to individual, is necessary for the effectiveness of G4H in people with psychosis.

G4H has been adapted for a range of populations and life changing contexts. Adapted versions for veterans, people who have experienced a brain injury and for children (to build social capability and prevent mental health decline) are in various stages of development and piloting. There is also evidence speaking to the efficacy of adapted versions for retirement, both from the workforce (La Rue et al., 2024), and elite sport (Haslam et al., 2024; Young et al., 2024). Like the original program, these adaptations emphasise the importance of developing group memberships and social identities to support health and wellbeing in the context of adjusting to challenging contexts or life events. Most, but not all (GROUPS 4 RETIREMENT and MORE THAN SPORT are both online programs primarily for individual delivery), are delivered in a group context, to take advantage of the group context and support from others with similar lived experience, which is argued to be a core mechanism of change in the program (e.g. Haslam, Cruwys, Haslam, Dingle, & Chang, 2016). Nevertheless, the use of a group setting can be an obstacle for some people. In the clinical context, clients with psychosis are often prepared to join general groups, but not necessarily a therapeutic group given past histories of difficult relationships including experiences of abuse and trauma and related issues of trust. Having the individual option potentially makes the intervention more acceptable and accessible, provided it is feasible and has some indication of effectiveness. It may be that similar outcomes in terms of identity integration could be achieved in group and individual delivery. For this reason, we consider that the inclusion of both individual and group formats is a strength of this study.

### The current study

The purpose of this feasibility randomised controlled trial was first to test the feasibility and acceptability of delivering the G4H intervention for loneliness to people with psychosis. The second purpose was to provide a preliminary comparison of the original group‐based format with an individual format developed specifically for this study. G4H has not previously been adapted for either psychosis or individual delivery. Given the social and socio‐cognitive challenges associated with psychosis, the individual format may be preferable. Thus, the current study serves as a pilot study for the adaptation of the intervention not only to psychosis but also to an individual format, primarily targeting acceptability and feasibility. Measures of key outcome variables of loneliness and wellbeing are included to explore their acceptability. Similarly, measures of possible mechanisms of change are included primarily to test acceptability. These include: social identification as someone who has unusual beliefs and experiences, the integration of this identity with other social identities, and perceptions of empathy, in both in‐group and outgroup members.

## METHOD

### Participants and design

The present study is a mixed methods feasibility trial, conducted between December 2022 and July 2024. Ethical approval was provided by the NHS Integrated Research Application System IRAS and Health Research Authority (project ID: 286005). The trial was pre‐registered with the Open Science Framework (DOI 10.17605/OSF.IO/Q973M). The study is reported in accordance with the CONSORT 2010 statement: extension to randomised pilot and feasibility trials (Eldridge et al., 2016).

Individuals were recruited from early intervention for psychosis services, adult mental health teams, local recovery colleges and charities. Inclusion criteria were: self‐identified psychosis (formal diagnosis was not required) and loneliness, able to communicate in English, aged 18–65 years, and able to give informed consent. Exclusion criteria included: inpatient status or being too unwell at the time of recruitment to be able to participate, primary drug or alcohol diagnosis, unwillingness to be randomised or to participate in either an individual or group intervention, unable or unwilling to travel to sessions, and unable to commit the time. Health service, recovery college staff or charity coordinators made first contact. Individuals were then screened over the telephone by the first author, an experienced clinical psychologist or a lived experience researcher, and those eligible were seen in person by these research team members together within 2 weeks of the start of the intervention. The first author established through clinical interview that participants had past or present experience of psychotic symptoms. All willing participants provided written informed consent and completed baseline questionnaires regarding socio‐demographic characteristics and measures of loneliness, wellbeing and social identity related variables. Socio‐demographic and clinical characteristics are presented in Table 1, and reflect that participants randomised to the different G4H formats were generally similar.^2^

**TABLE 1:** Socio‐demographic and clinical characteristics of participants allocated to individual (*n* = 20) and group (*n* = 20) format interventions.

Variable	Intervention format	
	Individual	Group
Age (years)		
Mean	38.75	43.65
Range	19–58	19–61
*SD*	12.10	12.52
Gender		
Male	9	7
Female	11	12
Prefer not to say	0	1
Ethnicity		
White British	14	15
White other	3	3
Indian	1	0
Pakistani	1	0
Bangladeshi	1	0
Black Caribbean	0	1
Mixed White and Asian	0	1
Employment status		
Employed (Full time)	4	2
Employed (Part time)	4	4
Unemployed (available for work)	1	0
Not able to work/on benefits	9	9
On sick leave	0	1
Student	1	2
Retired	0	1
Other	1	1
Living Circumstances		
Family	7	9
Living alone	10	8
Sharing with strangers	2	2
Other	1	1
Recruitment source		
Adult Mental Health Team	5	6
Early Intervention Service	2	3
Recovery College	8	8
Bipolar UK	5	3
Time since psychotic experiences started (years)		
Mean	16.26	16.20
Range	1–53	0–52
*SD*	13.79	14.92
Time since first diagnosed (years)		
Mean	8.12	14.32
Range	0.50–33.00	0–57.00
*SD*	9.08	15.70
Duration of NHS contact (years)		
Mean	11.00	12.73
Range	0.50–33.00	0.33–40.00
*SD*	10.73	12.04
NHS continuing contact		
Yes	13	14
No	7	5
Missing	0	1
Currently taking Medication		
Yes	16	13
No	3	6
Missing	1	1
Diagnosis		
Schizophrenia	4	4
Acute and Transient Psychotic Disorder	4	3
Schizoaffective Disorder	0	1
PTSD	1	1
Bipolar Disorder	3	3
Other	0	2
Never given a diagnosis	1	1
Multiple diagnoses with psychosis	1	4
Multiple diagnoses without psychosis	6	0
Missing	0	1
Number of past hospital admissions		
Mean	0.26	0.68
Range	0–1	0–9
SD	.45	2.08
Previous treatment for loneliness		
Yes	2	0
No	18	19
Missing	0	1
Currently receiving psychological therapy		
Yes	4	3
No	16	16
Missing	0	1

*Note*: NB: Data is only included for participants who completed each variable.

Participants were randomly allocated to individual or group formats of G4H. Allocations were made on a ratio of 1:1 and in blocks of four, by an administrator using the computerised system Sealed Envelope (https://www.sealedenvelope.com). Baseline measures were repeated at end of treatment (session 4), 1 month follow‐up (session 5) and 6‐month follow‐up. All assessments were carried out in‐person by the first author who also conducted the interventions, and/or the lived experience researcher who co‐delivered the group intervention. Measures were completed at the end of the relevant session and returned by hand. The only exception to this was at 6‐month follow‐up when questionnaires were posted to participants with a stamped addressed envelope to be returned by post. Consistent with the CONSORT 2010 statement: extension to randomised pilot and feasibility trials (Eldridge et al., 2016) measures of dependent variables were primarily included to test measurement feasibility; however, exploratory analyses were also conducted to inform a future full trial design. All participants were reimbursed £10 for their time spent on each occasion of completing questionnaires.

### G4H intervention

The group and individual formats followed the G4H treatment manual (Haslam, Cruwys, Haslam, Bentley, et al., 2016b) and workbook (Haslam, Cruwys, Haslam, Bentley, et al., 2016c). The intervention contains five modules (schooling, scoping, sourcing, scaffolding and sustaining). Participants first learn about the importance of social groups to wellbeing. They then reflect on important self‐aspects and map out their existing social networks following the social identity mapping (SIM) protocol developed by Cruwys et al. (2016). They reflect on how they relate to their groups (e.g. goodness of fit) and the resources their groups might be able to provide (e.g. support, enhancing positivity and self‐esteem) and how their groups relate to each other (i.e. their (in)compatibility). Finally, participants consider how to make the most of their existing social groups and make an individualised social plan involving nurturing existing, reconnecting with previous and/or joining new social groups. At 1‐month follow‐up, social plans are reviewed and revised where needed to be more effective. In the traditional group delivery format, the group acts as a support and resource with an overall objective of facilitating a positive experience of social connection within the group and using this as a platform to build social connections within the local community. Four groups were run in succession, each with 4–6 participants at the outset. Group size was kept small to allay anxiety and avoid long delays to starting. Group sessions were co‐delivered with a lived experience researcher. This researcher, with support from a trial participant, also developed a directory of social groups to support participants in both arms of the trial to identify local groups that might meet their social needs. The individual format followed the same protocol as the group format but was delivered 1:1 by the first author. The G4H protocol suggests a check‐in with group leaders mid‐way between sessions 4 and 5. In‐person check‐ins as a group were offered to those in the group format arm of the trial. Fourteen of the 16 participants who completed the group format took up the offer of a check‐in session, 3 of these were online, consistent with participant preference. Participants in the individual format arm of the trial were offered individual check‐ins, either in‐person or online. Nine of the 18 participants who completed individual treatment opted for an online check‐in and the other 9 in‐person. Thus, the intervention consisted of five formal sessions spaced over 2 months, and one informal check‐in, that is, six sessions in total. The first author attended two workshops delivered by the G4H development team and was supervised by the third author, the developer of G4H, on a fortnightly basis throughout the running of the trial. Both formats were delivered in‐person in a hospital outpatient clinic. A fidelity measure developed for G4H was used to ensure all sessions covered key topics/activities.

The G4H manual was adapted to make it more relevant for people who experience psychosis. Adaptations were co‐developed with a lived experience advisory panel (LEAP) comprising eight people with psychosis. The main protocol adaptations for psychosis (proposed by the LEAP) were:

- Longer sessions (group‐2 h with breaks; individual‐1 h).
- Greater focus on discussion over writing in workbooks with session summaries and homework tasks emailed to participants after sessions.
- Example social identity maps were shared for reference, and concepts such as identity compatibility clarified.
- Acknowledgement and normalising impact of symptoms, diagnosis and medication on group memberships.
- Consideration of psychosis‐related barriers to social connection.
- Extension of challenging groups segment to include reflection on stigma (including self‐stigma) and discrimination experienced in psychosis.
- Support with disclosure decision‐making and conversational skills.

In addition to the above adaptations, some further adjustments were made in the course of the study in response to individual need. The reflection task after session 1 in which individuals are asked to note down challenges experienced in the past and the different ways their social groups could have supported was distressing for some who had past histories of abuse and bullying. This exercise was adapted by asking participants to consider the kind of support they would like from their social groups in the future. In developing social maps in session 2, participants were encouraged to note all important social groups including groups of one person and the one could be a pet or even in one case a ‘voice’. In session 3, some participants found it distressing to consider how their social maps had come to be as they were, as for some this reflected loss of important relationships with the advent of mental health challenges. Rather than a specific adjustment, this stimulated helpful discussion of stigma, prejudice and discrimination and the difficulties surrounding disclosure‐decision‐making. Trial therapists took the position within such discussions of supporting individuals to make their own disclosure‐decisions rather than advocating a particular course of action. Many participants found it helpful to reflect on why social groups had been lost and whether they could have done more to nurture their groups, and also whether there was scope for rekindling these. Consistent with this, some sessions, both group and individual, included reflection on, and practice of, social skills such as starting conversations, turn‐taking, and active listening. Most participants found it empowering to evaluate their groups and this often led to discussion about toxic groups that were often family‐based but could include those that promoted unhelpful behaviour particularly for people with psychosis such as drug and alcohol use. Participants were encouraged to make strategic decisions regarding continued investment in such groups. In session 4, it was not always possible to develop SMART goals for everyone involving joining a new group and strengthening an existing group, however, everyone had at least one SMART goal and care was taken to ensure that this was appropriate and achievable in order to minimise risk of failing. Across all sessions, a proactive approach was taken towards attendance given motivational issues and reduced activity levels associated with psychosis; individuals were asked about anticipated challenges and support needed. In response to this, repeated prompts were delivered via emails, letters, and phone calls as requested and in some cases family members and health professionals were enlisted to transport participants to and from appointments and social groups.

The trial was supported by a steering committee comprising service leads for all recruitment sites and representatives of the LEAP. LEAP members were reimbursed (£10 per hour) for their contribution.

### Changes from pre‐registered protocol

A few changes were made to the protocol pre‐registered with OSF. These are as follows:

1. The option of group or individual sessions exclusively online was withdrawn as there were insufficient participants who preferred this format at any one time to be able to run a group after randomisation. Also, the G4H intervention was not adapted for online delivery at the point of running the trial. On reflection, it was also considered that introducing this option would interfere with the stated aims of the trial being to test the feasibility of a version adapted for psychosis and individual format delivery.
2. The original intention was to include participants who self‐reported loneliness and also had 3 or fewer social contacts in the previous week (excluding people in their workplace, health care staff or people they live with) consistent with the SCENE 5 protocol. However, participants in our study found it difficult to recall this information accurately and make these differentiations. As a result we disregarded the social contacts data and included people who self‐reported loneliness and met other inclusion criteria.
3. The individual intervention was initially planned to follow the SCENE 5 protocol, however, it was later decided that it would make for a better comparison to compare the usual G4H group format with a one‐to‐one (i.e. client‐therapist) delivery version. Thus, the one‐to‐one intervention consisted of five sessions, rather than the six of SCENE, with sessions held weekly, as opposed to monthly, and lasting a full hour rather than the minimum duration acceptable for follow‐up sessions within SCENE of 20 minutes. The workbook, session content, and activities, including between‐session tasks, were all consistent with the original G4H program also, as opposed to SCENE.

### Measures

**Trial feasibility** was measured at 1‐month follow‐up using an adapted version of the scale developed for, and used in, the imagery‐based emotion regulation trial (IBER) (Steel et al., 2023). This assessed experiences of trial procedures and interventions including satisfaction with information provided, eligibility assessment, questionnaire clarity, time to complete questionnaires, reminders to complete questionnaires, and overall trial satisfaction (all rated on a scale from 1 ‘not at all satisfied’ to 5 ‘extremely satisfied’). Participants were also asked about their preferred format (i.e. whether the same as that they received or different) and their satisfaction with the length of the intervention (response options included ‘too short’, ‘about right’ and ‘too long’).

The following self‐rated outcome measures were completed at baseline (within 2 weeks of the first session), end of treatment (end session 4), 1‐month follow‐up (end session 5) and 6‐month follow‐up:

**Loneliness** was measured with the University of California Los Angeles (UCLA) Loneliness Scale (ULS‐8), (Hays & Dimatteo, 1987), a short revised version of the UCLA loneliness scale that has demonstrated validity and internal reliability (Cronbach's alpha 0.84) (Wu & Yao, 2008). The scale comprises eight statements rated on a 4‐point scale of 0 ‘I never feel this way’ to 3 ‘I often feel this way’. An example item is ‘I lack companionship’. The scale has two reverse scored items, an example of which is: ‘I am an outgoing person’. Higher scores indicate greater loneliness. Cronbach's alpha for the current study for the ULS‐8 ranged from 0.63–0.85 across time points 1–4.

**Wellbeing** was measured using the Warwick‐Edinburgh Mental Wellbeing Scale (WEMWBS) (Tennant et al., 2007). This scale comprises 14 statements; each scored from 1 ‘none of the time’ to 5 ‘all of the time’ in relation to experience over the preceding 2 weeks. Sample items include ‘I've been feeling useful’, and ‘I've been feeling loved’. Higher scores reflect better wellbeing. The scale has good internal consistency (Cronbach's alpha 0.89 in a student sample and 0.91 in a general population sample), content and criterion validity and test–retest reliability (Tennant et al., 2007). Cronbach's alpha for the current study was 0.81–0.91 across time‐points 1–4.

**Social identification** was measured using a 14‐item scale based on the hierarchical multi‐component model of in‐group identification (IGI) developed by Leach et al. (2008). Each statement is scored from 1 ‘strongly disagree’ to 7 ‘strongly agree’ with higher scores indicating greater identification. Wording of statements was adapted to refer to the target in‐group as a group of people who have ‘unusual beliefs and experiences’; all other wording was as in the original.^3^ Sample items include ‘I feel a bond with other people who have unusual beliefs and experiences’ and ‘The fact that I have unusual beliefs and experiences is an important part of my identity’. The measure has good internal consistency (Cronbach's alpha was 0.80–0.93 across European and Dutch general population samples), construct, predictive and discriminant validity (Leach et al., 2008). Cronbach's alpha for the current study was 0.83–0.92 across time‐points 1–4.

**Identity Integration** was measured using the integration subscale of the Multi‐cultural Identity Integration Scale (MULTIIS) with wording adapted to refer to ‘unusual beliefs and experiences’ instead of cultural identities; all other wording was the original (Yampolsky et al., 2016). The subscale has eight statements each rated on a 1–7 scale from 1 ‘Not at all’ to 7 ‘Exactly’. Higher scores indicate greater integration. An example item is ‘My identity as someone who has unusual beliefs and experiences fits within a broader identity’. The original MULTIIS has good internal consistency (Cronbach's alpha of 0.85 for the integration subscale), and convergent and predictive validity (Yampolsky et al., 2016). Cronbach's alpha for the current study was 0.79–0.85 across time‐points 1–4.

**Perceived empathy** from both in‐group (i.e. people with psychosis) and outgroup members (i.e. people who do not have disclosed psychosis) was measured using appropriately adapted versions of the Perceived Empathy Scale (PES) (Nambisan, 2011). Participants rated from 1 ‘Not at all’ to 7 ‘totally’ how well eight different statements reflected their perception of others. Section 1, perceived empathy scale for psychosis (PESP) related to perceptions of people who have disclosed unusual beliefs and experiences, and section 2, perceived empathy scale for people with no psychosis (PESNP) related to perceptions of other people who have not disclosed unusual beliefs and experiences. Both scales have identical items; examples include ‘sympathetic’ and ‘supportive’. Higher scores indicate greater empathy. The scale has one factor and good internal consistency (Cronbach's alpha was 0.95 across 3 online health communities for physical health issues) (Nambisan, 2011). Cronbach's alpha for PESP in the current study was 0.90–0.96 across time‐points 1–4, and for PESNP 0.94–0.95.^4^

In addition to the above scales completed at four time points, social networks were assessed using SIM^5^ in sessions 2 and 5, as per the G4H protocol (Cruwys et al., 2016).

Therapists checked in with participants at each therapy session for both formats to monitor mental health. Adverse events were defined in the trial as any self‐reported worsening of mental health related to trial participation, and serious adverse events as hospital admissions or suicide attempts. Participants consented at the outset of the trial to health professionals involved in their care being contacted in such events.

### Analytic strategy

The primary purpose of the study was to test the feasibility and acceptability of the individual and group versions of the G4H intervention. Differences between scores for participants receiving the individual and group formats were tested using t‐tests and chi square analyses. Exploratory repeated measures ANOVAs were conducted to examine changes over time on the key dependent variables of loneliness, wellbeing, and the potential mechanisms of in‐group identification, identity integration, and perceived empathy, both of in‐group and outgroup, with intervention format as a between‐subjects factor. Paired t‐tests were used to examine change over time between baseline, end treatment and 1 month follow up in the key dependent variable of loneliness. All statistical analyses were conducted using SPSS (version 29.0). Means were used to replace missing values assuming no more than 20% or 2 per questionnaire per participant. Scores were excluded for participants with more than 2 missing values. There was little variation in missing data across time points and formats. Taking account of drop‐outs, and excluding follow‐up data at T4, the largest number of missing questionnaires across time points and formats was 8 for identity integration, followed by 6 for both perceived empathy in people with psychosis and those without psychosis, and 4 for loneliness, wellbeing, and in‐group identification. The main treatment outcome analyses were based on intention to treat (ITT) (McCoy, 2017) using the Last Observation Carried Forward Strategy. Although there are some potential limitations of the LOCF approach (Gadbury et al., 2003; Lane, 2008), it is more conservative than other approaches and therefore more suited to an initial feasibility trial. Also, it does not rely on the assumption that data is missing at random in the way that imputation does. Per protocol (PP) figures are also specified in Table 3. PP excludes data for participants who withdrew. ITT analyses excluded participants for whom baseline data was missing.

## RESULTS

### Trial feasibility

#### Recruitment and retention

**FIGURE 1:**
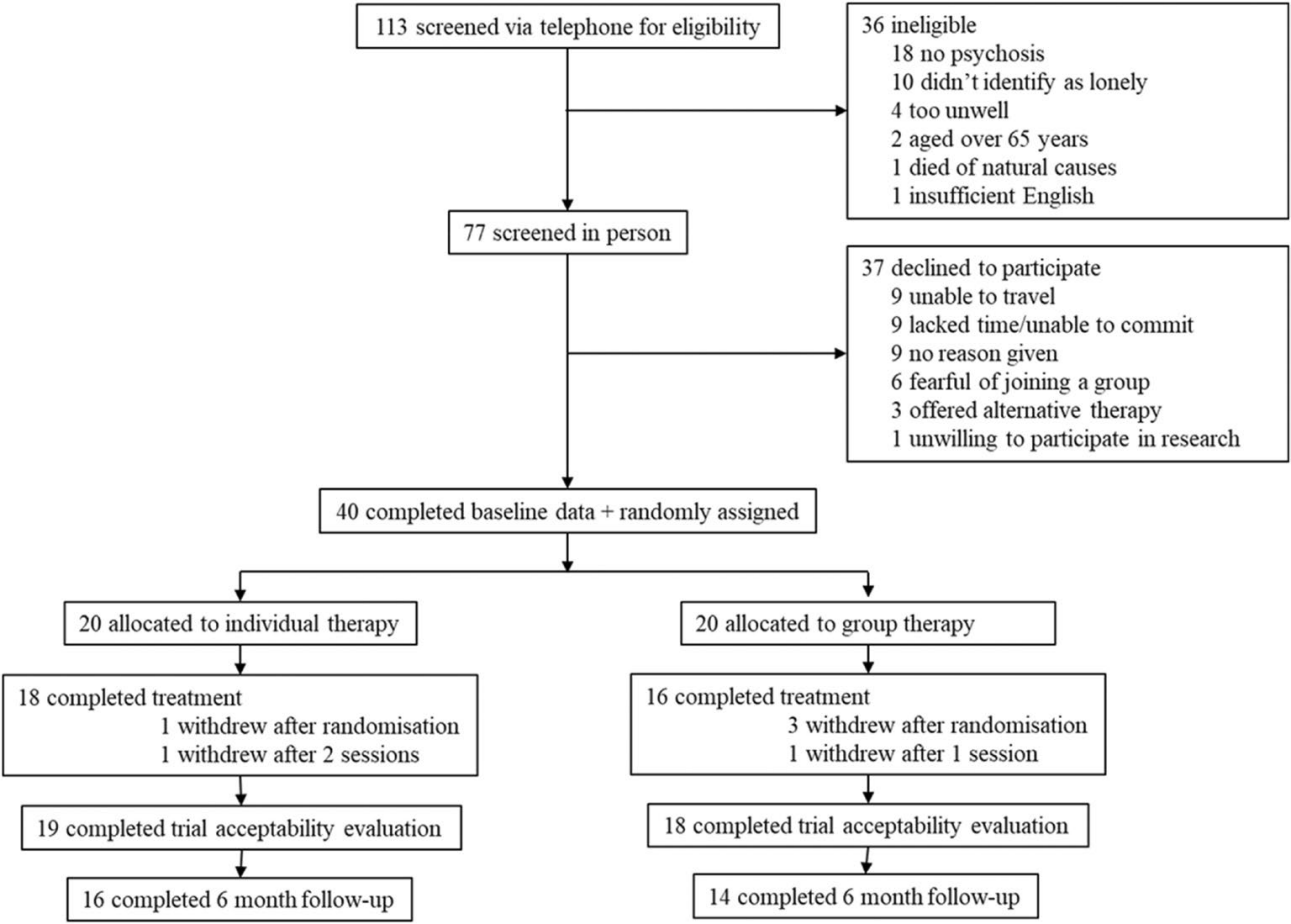
Consort diagram of trial profile.

The trial recruited 40 participants (125% of target) over a 9‐month period (see CONSORT diagram, Figure 1). The initial recruitment target was 32, however this was later increased to 40 to compensate for drop‐outs. Four participants dropped out after randomisation: one allocated to the individual intervention because they preferred group, 3 allocated to group, two due to being offered another therapy and one gave no reason. In line with the G4H protocol, participants were considered to have received an adequate dose of therapy if they received a minimum of 3 sessions out of 5 and received catch up sessions to ensure continuity across sessions. One participant from each arm of the trial withdrew without reaching this criterion and in both cases, this was due to deterioration in mental health verbally reported to trial staff (according to participants' report, this was not related to trial participation in either case). Of the 18 participants who completed the group arm of the trial, 4 needed a catch‐up session and one participant needed 2 catch‐up sessions. Of the 16 participants who completed the individual arm of the trial, all sessions were ultimately attended but 4 participants needed to have one missed session rescheduled and 2 participants needed to have 2 missed sessions rescheduled. In total, 34 (85%) participants accepted into the trial completed treatment (Individual *n* = 18, Group *n* = 16). There was some disengagement with research assessments as can be seen in Tables 2, 3, 4. Looking at the pattern of missing data on specific measures, all seem acceptable. The greatest amount of missing data was found for the measure of identity integration. Participants fed back to trial therapists that they found the concept of identity integration and associated scale items particularly difficult to understand. Future research should further investigate the clarity and appropriateness of the measure of identity integration for this population. There were no serious adverse events reported.

**TABLE 2:** Trial acceptability ratings by intervention format at 1‐month follow‐up.

Variable	Intervention format							
	Individual				Group			
	Mean	*SD*	*N*	(95% CIs)	Mean	*SD*	*N*	(95% CIs)
Information provided	4.37	0.90	19	(3.94–4.80)	4.28	1.13	18	(4.72–4.84)
Eligibility assessment	4.32	0.89	19	(3.89–4.74)	4.24	0.97	17	(3.74–4.73)
Questionnaire clarity	3.58	1.12	19	(3.04–4.12)	3.94	1.11	18	(3.39–4.50)
Time to complete questionnaires	3.84	1.02	19	(3.35–4.33)	3.83	1.10	18	(3.29–4.38)
Questionnaire reminders	4.06	0.72	18	(3.69–4.42)	4.29	0.69	17	(3.94–4.65)
Overall trial satisfaction	4.37	0.68	19	(4.04–4.70)	4.11	1.23	18	(3.50–4.72)
Satisfaction with randomisation	4.37	0.90	19	(3.94–4.80)	4.11	0.90	18	(3.66–4.56)
Preferred treatment after delivery	(*n*= 18)				(*n*=17)			
Same as randomised (%)	14 (70%)				12 (60%)			
Different to randomised (%)	4 (20%)				5 (25%)			
Satisfaction with treatment length	(*n*=19)				(*n*=18)			
Too short (%)	4 (20%)				4 (20%)			
About right (%)	15 (75%)				13 (65%)			
Too long (%)	0 (0%)				1 (5%)			

*Note*: NB: All ordinal scales were scored from 1 ‘not at all satisfied’ to 5′ extremely satisfied’.

#### Trial acceptability

Means and confidence intervals for all acceptability variables in relation to trial format (individual or group) are presented in Table 2.

All 40 eligible participants were invited to complete the trial acceptability questionnaire at session 5, the end of the active intervention period. Nineteen (95%) participants in receipt of the individual format completed this and 18 (90%) from the group format. Mean scores for all variables and both formats were above the midpoint of 3 for the 1–5 scale (corresponding to ‘moderately’ to ‘extremely satisfied’). As can be seen in Table 2, Means, SDs and 95% confidence intervals suggest participant satisfaction with information provided about the trial, the eligibility assessment process, reminders to complete questionnaires, and randomisation. The majority of participants reported satisfaction with the intervention they were assigned to (70% for individual and 60% group). The majority of participants in both formats were also satisfied with the length of treatment (75% for individual and 65% group) with most of those preferring a different length expressing the view that the intervention was ‘too short’.^6^

### Outcome variables

Descriptive statistics for outcome scores are presented in Table 3. Data are presented for all four time points including T4, 6‐month follow‐up, however, only data for time‐points 1–3, the active intervention phase are used in exploratory analyses given sample attrition. The mean total loneliness score at baseline T1 for the primary dependent variable, ULS‐8 loneliness, and ITT data was 17.30 (SD 3.25) for those who had the individual format and 17.42 (SD 3.17) for those who had the group format. These ULS‐8 loneliness scores are broadly similar to those reported by Alasmawi et al. (2020) in a sample of 106 people diagnosed with psychosis and accessing care from UK secondary mental health services (Mean 19.69, SD 4.89).

**TABLE 3:** Means and confidence intervals by format and time point for intention to treat (ITT), and per protocol (PP) figures.

Variable	Baseline (T1)		End treatment (T2)		1‐month follow‐up (T3)		6‐month follow‐up (T4)^a^	
	Mean (*N*)	*SD*	Mean (*N*)	*SD*	Mean (*N*)	*SD*	Mean (*N*)	*SD*
Loneliness ULS‐8								
Individual (PP)	17.00 (17)	3.45	15.53 (17)	3.26	13.18 (17)	2.68	13.44 (16)	5.15
(95% CI)	(15.28–18.72)		(13.74–17.32)		(11.35–15.00)		(10.69–16.18)	
Individual (ITT)	17.30 (20)	3.25	15.90 (20)	3.18	13.60 (20)	2.84	13.75 (20)	4.79
(95% CI)	(15.85–18.75)		(14.33–17.47)		(11.91–15.29)		(11.51–15.99)	
Group (PP)	17.13 (15)	3.48	15.47 (15)	3.96	14.00 (15)	4.57	14.64 (14)	4.60
(95% CI)	(15.31–18.96)		(13.56–17.39)		(12.06–15.94)		(11.99–17.30)	
Group (ITT)	17.42 (19)	3.17	16.11 (19)	3.76	14.95 (19)	4.48	15.63 (19)	4.30
(95% CI)	(15.85–18.75)		(14.49–17.72)		(13.22–16.68)		(13.56–17.70)	
Wellbeing WEMWBS								
Individual (PP)	37.24 (17)	8.69	41.00 (17)	6.05	44.35 (17)	8.18	43.19 (16)	8.86
(95% CI)	(32.90–41.58)		(37.66–44.34)		(40.25–48.45)		(38.47–47.91))	
Individual (ITT)	37.15 (20)	8.55	40.20 (20)	6.44	43.55 (20)	7.84	42.75 (20)	8.16
(95% CI)	(33.22–41.09)		(36.66–43.74)		(39.40–47.70)		(38.93–46.57)	
Group (PP)	36.13 (15)	8.85	42.27 (15)	7.47	45.13 (15)	8.39	43.14 (14)	10.06
(95% CI)	(31.51–40.75)		(38.71–45.83)		(40.79–49.50)		(37.33–48.95)	
Group (ITT)	34.68 (19)	8.83	39.53 (19)	9.04	41.79 (19)	10.38	40.53 (19)	10.93
(95% CI)	(30.65–38.72)		(35.89–43.16)		(37.53–46.05)		(35.26–45.79)	
In‐Group Identification Scale IGI								
Individual (PP)	50.41 (17)	13.30	58.35 (17)	14.77	57.65 (17)	14.13	55.19 (16)	12.73
(95% CI)	(43.83–57.00)		(50.40–66.31)		(49.87–65.42)		(48.40–61.97)	
Individual (ITT)	50.50 (20)	12.38	57.70 (20)	13.83	57.40 (20)	13.03	54.55 (20)	11.87
(95% CI)	(44.71–56.29)		(50.71–64.69)		(50.66–64.14)		(48.99–60.11)	
Group (PP)	57.27 (15)	13.25	62.67 (15)	17.41	59.93 (15)	17.32	59.14 (14)	22.15
(95% CI)	50.26–64.27)		(54.20–71.13)		(51.66–68.21)		(46.36–71.93)	
Group (ITT)	56.32 (19)	13.19	60.58 (19)	16.93	58.42 (19)	16.62	57.53 (19)	19.89
(95% CI)	(50.38–62.26)		(53.41–67.75)		(51.50–65.34)		(47.94–67.12)	
Identity Integration Scale IIS								
Individual (PP)	25.33 (15)	10.22	29.93 (15)	8.37	34.47 (15)	10.06	32.94 (16)	8.80
(95% CI)	(20.01–30.66)		(25.70–34.17)		(29.16–39.77)		(28.25–37.63)	
Individual (ITT)	24.94 (17)	10.26	27.76 (17)	9.94	32.12 (17)	11.58	31.05 (19)	10.00
(95% CI)	(20.08–29.81)		(23.16–32.37)		(26.74–35.04)		(26.23–35.87)	
Group (PP)	28.15 (13)	9.80	33.46 (13)	7.51	32.54 (13)	9.90	31.00 (14)	10.40
(95% CI)	(22.43–33.87)		(28.91–38.01)		(26.84–38.23)		(25.00–37.00)	
Group (ITT)	26.84 (19)	9.52	30.68 (19)	8.76	30.53 (19)	10.26	29.05 (19)	10.44
(95% CI)	(22.24–31.44)		(26.33–35.04)		(25.44–35.61)		(24.02–34.08)	
Perceived Empathy Scale‐Psychosis PESP								
Individual (PP)	40.06 (16)	13.04	40.25 (16)	13.28	40.31 (16)	12.62	37.13 (15)	11.57
(95% CI)	(34.16–45.96)		(33.73–46.77)		(33.42–47.20)		(30.73–43.54)	
Individual (ITT)	40.05 (19)	12.92	40.21 (19)	13.13	40.63 (19)	12.23	38.65 (20)	11.12
(95% CI)	(34.80–45.31)		(34.54–45.88)		(34.70–46.57)		(33.44–43.86)	
Group (PP)	41.83 (12)	8.92	40.25 (12)	13.28	40.30 (12)	12.62	38.14 (14)	14.03
(95% CI)	(35.02–48.64)		(37.80–52.87)		(30.46–46.37)		(30.04–46.24)	
Group (ITT)	43.06 (17)	9.07	45.53 (17)	10.96	41.00 (17)	13.27	40.00 (19)	13.40
(95% CI)	(37.50–48.62)		(39.54–51.52)		(34.72–47.28)		(33.54–46.46)	
Perceived Empathy Scale‐Non‐Psychosis PESNP								
Individual (PP)	28.29 (17)	12.67	30.18 (17)	10.16	32.76 (17)	10.68	28.94 (16)	10.18
(95% CI)	(22.46–34.19)		(24.62–35.73)		(27.21–38.32)		(23.52–34.36)	
Individual (ITT)	28.53 (19)	12.46	29.53 (19)	9.86	31.95 (19)	10.41	29.26 (19)	9.99
(95% CI)	(23.34–33.72)		(24.14–34.91)		(26.78–37.12)		(24.45–34.08)	
Group (PP)	26.62 (13)	10.38	32.08 (13)	12.41	28.69 (13)	11.81	27.79 (14)	10.57
(95% CI)	(19.94–33.29)		(25.73–38.43)		(22.34–35.04)		(21.68–33.89)	
Group (ITT)	24.16 (19)	9.66	27.05 (19)	13.07	24.89 (19)	11.76	26.16 (19)	10.83
(95% CI)	(18.97–29.35)		(21.67–32.40)		(19.73–30.06)		(20.94–31.38)	

^a^ 6‐month follow up T4 figures, are for all participants who completed questionnaires at this stage; only T1, T2 and T3 data are included in the exploratory ANOVA ITT and PP analyses. Some figures are higher at T4 due to missing earlier data.

**FIGURE 2:**
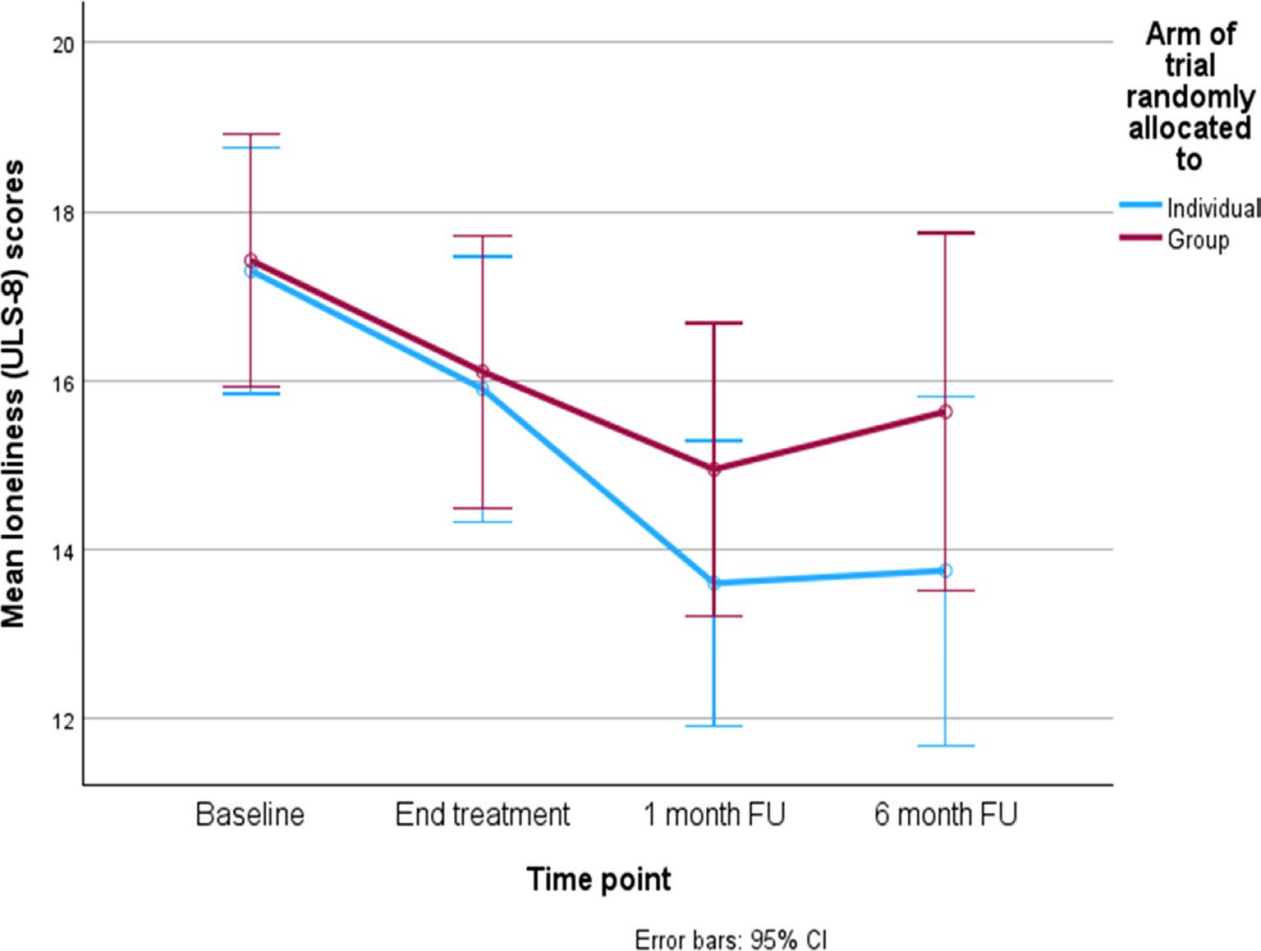
Mean loneliness (ULS‐8) scores between formats and across time points.

Repeated measures ANOVAs revealed no between format differences for the main treatment outcome variable of loneliness, using intention to treat analysis (ITT). However, there was significant reduction in loneliness scores over time, from baseline to 1‐month follow‐up, irrespective of intervention format, *F*(2,74) = 20.45, *p* < .001^7^ ^8^. See Figure 2 for the mean loneliness scores over time for both groups. Paired samples t‐tests were conducted to compare means across time‐points. The t‐statistic for T1–T2 was *t*(39) = 3.11, *p* = .003, for T1–T3 *t*(39) = 5.49, *p* < .001 and for T2–T3 *t*(39) = 3.89, *p* < .001. These results are all significant with Bonferroni correction and indicate that loneliness ITT scores at all time‐points are significantly different from each other indicating stepwise improvement in loneliness across time for both formats. In the ITT analysis, the mean differences from baseline to 1 month follow up on loneliness were 3.7 for the individual format, and 2.47 for the group; the baseline SD was 3.2, suggesting similar effect sizes across groups and very small group differences. The effect size (Cohen's *d*) for change in loneliness scores across both formats (combined scores) from T1 to T3 was 0.86, indicating a large effect size.

Repeated measures ANOVAs for wellbeing, in‐group identification, and identity integration ITT variables also revealed no between format differences but significant change over time, irrespective of intervention format. Perceived empathy in others with psychosis and perceived empathy in those without psychosis did not show either between format or across time statistically significant change. Per protocol analyses were similar to ITT therefore only ITT are reported.^9^

### Social identity mapping

SIM data are presented in Table 4 for participants assigned to individual and group formats. These are presented for sessions 2 and 5, consistent with SIM protocol. Changes in SIM variables across time points were small and statistical analyses were not conducted, given the small number of participants and large number of variables providing insufficient power. However, as can be seen from means in Table 4, most SIM variables appeared to change in a positive direction across both formats. Total number of social groups, and number of multiple‐person, bespoke, representative, high contact, supportive, positive, and *supergroups* all increased over time for participants receiving both formats. Compatibility lines reflected positive change for both formats including an increase in compatible lines and reduction in incompatible, particularly for those receiving the group format.

**TABLE 4:** Social identity mapping descriptive data as a function of intervention format.

Variables	Intervention format							
	Individual				Group			
	Session 2 (*N*=18)		Session 5 (*N*=16)		Session 2 (*N*=16)		Session 5 (*N*=13)	
	M (range)	*SD*	M (range)	*SD*	M (range)	*SD*	M (range)	*SD*
Total number of groups	6.17 (3–10)	1.72	6.94 (4–13)	2.26	6.56 (3–12)	2.53	6.85 (4–12)	2.48
Mental health‐based groups	1.00 (0–4)	1.24	1.06 (0–5)	1.53	1.25 (0–4)	1.34	1.08 (0–3)	1.12
Single person groups	1.22 (0–6)	1.52	1.56 (0–7)	1.93	0.87 (0–4)	1.15	0.69 (0–4)	1.18
Multiple person groups	4.78 (3–7)	1.31	5.38 (2–8)	1.59	5.69 (3–12)	2.47	6.15 (4–12)	2.27
Generic groups	3.89 (2–9)	1.78	3.81 (1–11)	2.54	3.69 (2–7)	1.54	3.15 (0–7)	1.86
Bespoke groups	2.28 (0–5)	1.84	3.13 (1–6)	1.54	2.75 (0–8)	2.35	3.69 (2–8)	1.97
Number of representative groups	3.39 (0–6)	1.65	4.38 (1–8)	2.12	3.81 (0–9)	2.37	4.85 (2–10)	2.38
Number of high contact groups	4.33 (0–8)	1.82	4.81 (2–8)	2.04	4.00 (1–10)	2.25	4.62 (1–10)	2.43
Number of supportive groups	3.72 (1–9)	1.96	5.13 (2–11)	2.63	3.69 (0–9)	2.55	4.77 (2–10)	2.32
Number of positive groups	4.56 (1–8)	1.85	5.44 (2–12)	2.63	4.06 (1–10)	2.25	5.38 (3–10)	2.10
Proportion of positive groups (%)	77.39 (14–100)	28.52	76.31 (29–100)	22.58	63.59 (14–100)	28.51	78.69 (57–100)	12.97
Number of supergroups	1.17 (0–5)	1.50	2.38 (0–6)	2.03	0.93 (0–3)^a^	1.03	2.38 (0–5)	1.71
Number of compatibility lines	4.28 (1–9)	2.47	7.00 (2–30)	6.63	3.93 (0–7)^a^	2.15	4.23 (0–9)	2.52
Number of moderate compatibility lines	2.94 (0–10)	2.67	3.88 (1–9)	2.63	1.40 (0–6)^a^	1.84	1.85 (0–7)	1.99
Number of incompatibility lines	2.06 (0–6)	2.01	1.38 (0–6)	1.67	2.80 (0–8)^a^	2.40	0.69 (0–3)	1.03

*Note*: Mental health based groups = support groups and charities; single person groups = relationships with one other individual; multiple person groups = groups with more than one individual; generic groups are common groups such as family, work colleagues; bespoke groups = groups reflecting special interests such as astrology, pottery; representative groups = those scored >5 for how representative the participant feels of the group; high contact groups = groups engaged with more than 50% of a typical month; supportive groups = groups scored >5 for support received; positive groups = groups scored >5 for positivity about membership of the group; supergroups = number of groups that scored above the midpoint on quality indicators of positivity, representativeness and support, and that have a majority (i.e. over 50%) of compatible lines to other groups; Number of compatibility lines = number of straight or green lines connecting groups, number of moderate compatibility lines = number of wavy or amber lines connecting groups and number of incompatibility7 lines = number of jagged or red lines connecting groups.

^a^ *N = 15*.

## DISCUSSION

This randomised controlled trial demonstrated the feasibility and acceptability of delivering and evaluating an adapted version of G4H to address loneliness in people with psychosis and indicated the viability of an adapted individual format version. In terms of feasibility, recruitment of forty individuals by a small research team was possible over a 9‐month period. Withdrawal rates were low and 85% of those accepted into the trial completed a minimum of 3 of 5 sessions, that is, 18 (90%) receiving the individual format and 16 (80%) group. This compares favourably with other G4H trials; 87% of participants completed a randomised non‐inferiority trial by Cruwys, Haslam, Rathbone, et al. (2022), comparing G4H with cognitive‐behaviour therapy for young people experiencing depression and loneliness. Importantly, in the current trial individual and group formats were very similar in terms of feasibility and acceptability. It is reasonable to conclude that a fully powered trial would be an appropriate next step. No clear overall preference was found for G4H format, individual or group. However, six individuals declined to take part at the assessment for eligibility stage due to fear of joining a group. These participants cited reasons of low confidence, social anxiety and mistrust of others following past abusive experiences. This supports the effort to establish that an individual format is feasible and acceptable. It is particularly encouraging to note that the effect of individually delivered sessions in terms of the full range of outcome variables was similar to group. This suggests that the group format may not be crucial to the delivery of G4H in psychosis. Alternatively, it might indicate that the participant‐therapist alliance functioned in a similar way to the group as an effective mechanism of change. This theorised group process has been proposed by Lee et al. (2021). Further, Cruwys and colleagues have demonstrated across two studies, involving over 500 psychotherapy clients, that the therapeutic alliance mediated the relationship between social identification and positive therapy outcomes (Cruwys, Lee, et al., 2023).

In terms of the overall effect of G4H, there was a significant reduction in loneliness over time, from baseline to 1‐month follow‐up, irrespective of format. Other variables relating to the potential mechanisms of action of the intervention follow the same pattern; that is, wellbeing, in‐group identification and identity integration, with the means being consistently similar. However, it is important to be cautious in interpreting these statistical analyses given the trial was designed to test feasibility rather than between group differences and the sample size was correspondingly small.

The current study demonstrates that SIM is an acceptable and feasible measure for understanding social networks and evaluating change in these for people with psychosis. Changes appeared to be broadly similar and positive across formats; however, these findings should be interpreted with caution given the small sample size and, crucially, the lack of a treatment as usual (TAU) control. Change in raw scores indicated an increase over time for participants in both individual and group formats in total number of groups, and number of multiple‐person, bespoke, representative, high‐contact, supportive, positive and *supergroups*. Number of *supergroups* (i.e. those that were scored above the midpoint on the four quality indicators of positivity, representativeness and support and had a majority of compatible lines to other groups) has been found in previous research to be positively related to wellbeing and adjustment to life changes (Bentley et al., 2020). Compatible lines, that is, the measure of compatibility between social groups in the SIM data reflected an increase in compatibility and decrease in incompatibility between groups across sessions. Compatibility between groups is likely to be a moderator of identity integration (i.e. the more compatible social groups are, the more likely it is that individuals will be able to integrate the related social identities into a coherent and positive sense of self). This hypothesis could be tested in future research using longitudinal designs and path analysis. Taken together, these social identity mapping results suggest improved strategic management of groups rather than simply increasing social contacts or activities. Participants in both conditions worked to review and shape their social worlds to better manage toxic groups and ensure a better fit between groups and with their sense of self.

### Limitations and future directions

The current trial was designed to test acceptability and feasibility, the sample size was correspondingly small, and there was no TAU control group. It is therefore difficult to draw any firm conclusions about the efficacy of G4H for people with psychosis, despite participants in both formats improving in relation to loneliness scores over time.

Recruitment to this trial was slow, related to reliance on busy mental health staff to make the first approach. Recruitment sources were extended to the charitable sector and Recovery colleges to manage this. This had the effect that diagnosis could not be verified through checking mental health records for most participants. Eighteen of the 40 individuals recruited into the trial (45%) lacked a verifiable diagnosis of psychosis. A consequence of this is that it is not possible to conclude that the study reports on a sufficient test of the interventions within a formally diagnosed psychosis population. Unfortunately, those who reported no formal psychosis diagnosis were also unevenly distributed across conditions. Future trials should formally verify diagnosis and ensure a more even split between conditions. Further, participants found it difficult to accurately recall number of previous social contacts in the previous week and differentiate workplace, health care staff and people they live with from other social contacts. We were therefore unable to recruit and characterise the sample in relation to number of social contacts and unable to make comparisons between this population and the wider population of people with psychosis. However, self‐reported loneliness could be considered more important than any objective index of social isolation in terms of engaging with, and benefitting from, the intervention.

The current trial was conducted following lockdowns in the UK related to the Covid 19 pandemic. Most participants were very willing to meet in person having felt socially isolated during lockdown. However, nine participants screened in person declined to participate in the trial because they were unwilling or unable to travel to sessions. An online version of G4H may be preferable for some. This was under development at the time of the trial but not yet available. The relative acceptability of this over in‐person formats will be important to test in future research.

The acceptability and feasibility results presented here provide support for a definitive randomised 2‐arm controlled trial. In PICO terms, such a trial should extend the participant group to include others with severe mental health challenges to increase accessibility and test the efficacy of this adapted G4H with a TAU comparator and loneliness as the primary outcome variable. In the active treatment arm of the trial, participants could be offered their preference of individual or group format, given the results of this feasibility trial suggested no clear difference between formats. Possible mechanisms of change should be tested including social identification with others who have similar mental health challenges, and integration of this social identity with other social identities into a coherent self‐concept. Perceived empathy could also be tested given previous research suggesting association between perceived empathy in other voice hearers and the formation of a voice hearer social identity, and perceived empathy in those who do not themselves hear voices and the integration of a voice hearer social identity (Hogg et al., 2023). Change in dependent variables should be assessed between baseline and session 5 (1 month follow‐up). Number of sessions should remain as per the G4H manual. It may also be worth considering a protocol change to offer an individual session as a precursor to moving to group sessions or involving someone who has completed group treatment in the recruitment process to increase motivation and address any concerns.

## CONCLUSION

Here, we demonstrated the feasibility and acceptability of both group and individual formats of the G4H intervention to address loneliness in people with self‐identified psychosis. Having shown a statistically significant reduction in loneliness and improvements in wellbeing, social identification and identity integration, our results provide a strong basis for a definitive randomised controlled trial. However, given the comparable changes across the two formats, randomisation to TAU versus active treatment may be best followed by a design allowing patient preference within the allocation to active treatment condition for group or individual format.

## AUTHOR CONTRIBUTIONS

**Lorna I. Hogg:** Conceptualization; methodology; investigation; funding acquisition; writing – original draft; visualization; writing – review and editing; formal analysis; project administration; data curation; resources. **Laura G. E. Smith:** Writing – review and editing; supervision; resources; validation. **Catherine Haslam:** Conceptualization; methodology; validation; writing – review and editing; supervision; resources. **Lyndsay Coxhill:** Investigation; methodology; writing – review and editing; project administration; resources. **Tim Kurz:** Validation; writing – review and editing; supervision; resources. **Georgina Hobden:** Project administration; validation; writing – review and editing; resources. **Anthony P. Morrison:** Conceptualization; methodology; validation; writing – review and editing; formal analysis; supervision; resources; data curation.

## CONFLICT OF INTEREST STATEMENT

All authors declare that they have no conflicts of interest to declare.

## Supporting information


Data S1.

## Data Availability

The data that support the findings of this study are available on request from the corresponding author. The data are not publicly available due to privacy and ethical restrictions.

## Footnotes

^1^
SCENE is defined as *improving quality of life and health outcomes of patients with psychosis through a new structured intervention for expanding social networks*.

^2^
See Table S1 for statistics indicating no between‐group differences for ordinal variables of age, time since psychotic experiences started, time since first diagnosis, duration of NHS contact, and number of past hospital admissions.

^3^
This wording was used rather than “psychosis” in order to appeal to all participants and not deter those who might find medicalised terms off‐putting.

^4^
Cronbach's alphas for all dependent variables and all time points are presented in Table S2.

^5^
See Data S1 for the social identity mapping (SIM) protocol.

^6^
T‐statistics and chi square statistics are presented in Table S3.

^7^
Mauchly's tests were not significant for all ANOVAs indicating normality of distributions, regardless of time point, with the exception of wellbeing and social identification, therefore a Greenhouse–Geisser correction was used for analyses of these variables.

^8^
Homogeneity of variance was only significant at time point 3 (*p* = .045).

^9^
See, Table S4 for ITT ANOVAs and Table S5 for PP ANOVAs.
