# Supplementary material for: A randomised feasibility trial comparing group and individual format GROUPS FOR HEALTH interventions for loneliness in people who experience psychosis
Source: Psychol Psychother. 2025 Jan 29;98(2):478–500. doi: 10.1111/papt.12574 (PMC12065070; doi:10.1111/papt.12574)
Supplement: Supplementary file 1 — Data S1. [file PAPT-98-478-s001.docx]

**Supplementary materials**

**Exploratory Statistics**

**Table S1:**

*Test statistics for between format differences in demographic and clinical ordinal variables*

*t-statistic df p*

Age -1.26 38 0.22

Time since psychosis started (years) 0.01 36 0.99

Duration of Health Service contact (years) -0.48 36 0.64

Time since first given diagnosis (years) -1.49 36 0.15

Number of times admitted -.86 19.70 0.40

**Table S2:**

*Cronbach’s alpha for dependent variables across time points*

T1 T2 T3 T4

Loneliness (ULS-8) .637 .722 .676 .857

Wellbeing (WEMWBS) .890 .815 .895 .912

Social identification (IGI) .831 .877 .880 .922

Identity integration (IIS) .799 .838 .854 .822

Perceived empathy psychosis

(PESP) .901 .962 .956 .944

Perceived empathy non-psychosis

(PESNP) .941 .957 .943 .940

**Table S3:**

*Test statistics for between format differences for trial acceptability ratings*

*t-statistic χ^2^ df p*

Information provided 0.27 35 0.79

Eligibility assessment 0.26 34 0.80

Questionnaire clarity -1.00 35 0.33

Time to complete questionnaires 0.03 35 0.98

Questionnaire reminders -1.00 33 0.33

Overall trial satisfaction 0.79 35 0.43

Satisfaction with randomisation 0.87 35 0.39

Preferred treatment after delivery* - 0.10 1 0.92

Satisfaction with treatment length** - - - -

**With Yates continuity correction*

***Chi square not possible given cell sizes of<5 and 3x2 table*

**Table S4:**

*Analyses of Variance for Intention to Treat (ITT) within-subjects data (main effect of time, baseline to 1-month follow-up)*

F ƞ2

Loneliness (2, 74), 20.45** .36

Wellbeing (2, 74), 14.83** .29

In-group identification (2, 74), 5.23* .12

Identity integration (2, 68), 5.91* .15

Perceived empathy (psychosis) (2, 68), 1.37 .04

Perceived empathy (non-psychosis) (2, 72), 0.87 .02

* *p<.01*

***p<.001*

**Table S5:**

*Analyses of Variance for Per Protocol (PP) within-subjects data (main effect of time, baseline to 1-month follow-up)*

F ƞ2

Loneliness (2, 60), 19.02** .39

Wellbeing (2, 60), 15.72** .34

In-group identification (2, 60), 4.68* .14

Identity integration (2, 52), 7.09* .21

Perceived empathy (psychosis) (2, 52), 2.38 .08

Perceived empathy (non-psychosis) (2, 56), 1.76 .06

* *p<.01*

***p<.001*

**Social Identity Mapping (SIM) protocol**

Social identity mapping takes place in G4H at sessions 2 and 5. Participants are first supported to write the name of each of their current social groups on post-it notes (with choice of three sizes to denote relative importance). They then stick these post-it notes on a sheet of A3 paper (juxtaposition on sheet reflecting degree of similarity between groups).

Participants then evaluate each group on four variables, three of which are rated 1-10 (representativeness, support, and positivity) and the fourth (participation) is rated 0-30 depending on number of days they engage with the group in a typical month. Finally, participants rate the compatibility between their groups (i.e., how easy it is to be a member of both) using either straight, wavy or jagged lines to denote increasing incompatibility or, if preferred, coloured pens using a traffic light system of green, amber and red.

In the current trial, separate scores were compiled for total number of groups, and number of groups with each of representativeness, positivity, support, and participation scored above the scale mid-point of 5. The proportion of positive groups (i.e., those scoring above 5 to those scoring 5 or below) was calculated (given that change in this variable might better reflect improved strategic management of social groups than change in overall number of positive groups).

Totals were also calculated for each participant for number of groups involving 1-person, multiple people, and also number of general groups (e.g., family, friends, neighbours, work colleagues), bespoke groups (e.g., running, art, paintballing, astrology) and mental-health related groups (e.g., charities such as MIND and Bipolar UK). Number of compatible, moderately compatible and incompatible lines between groups for each participant at each of the two SIM assessment points was summed. The Number of *supergroups* (Bentley et al. (2019) was calculated, that is number of groups that scored above the midpoint on the four quality indicators: positivity, representativeness and support, and that had a majority (i.e., over 50%) of compatible lines to other groups. This construct is predictive of wellbeing and adjustment to life changes (Bentley et al., 2019).
